# Characterization of Shear Horizontal Waves Using a 1D Laser Doppler Vibrometer

**DOI:** 10.3390/s21072467

**Published:** 2021-04-02

**Authors:** Alaa Elhady, Eihab M. Abdel-Rahman

**Affiliations:** Department of System Design Engineering, University of Waterloo, Waterloo, ON N2L 3G1, Canada; alaaeldin.ahmed@edu.uwaterloo.ca

**Keywords:** SH waves, Bleustein–Gulyaev waves, vibrometer, SAW detection, MEMS characterization

## Abstract

We developed a new technique for the detection of shear horizontal surface acoustic waves (SH-SAW) using a one-dimensional laser-based Doppler vibrometer. It measures the out-of-plane surface deformation at the fingertip of an interdigitated transducer (the boundary of the wave aperture) and uses it to estimate the instantaneous in-plane displacement field given the substrate Poisson ratio. It can also estimate the degree of surface confinement (wave decay rate). The proposed approach was first verified using finite element analysis (FEA) and demonstrated experimentally using a Bleustein–Gulyaev resonator.

## 1. Introduction

Surface acoustic waves (SAWs) propagating in piezoelectric materials have numerous applications, such as in sensors [1,2,3], actuators [4,5], filters [6,7,8], delay lines [9], and radio-frequency identification applications [10,11]. They are usually excited via a set of interdigitated electrodes (IDTs) that are deployed as transmitters and receivers or as non-collocated [12,13] or collocated [14] transceivers. Resonant transceivers create standing waves by trapping the waves using reflectors made of another set of IDTs [14] or an acoustic impedance mismatch, such as deep grooves [15].

Characterization of these waves is integral to SAW technology and research. A common method for SAW characterization is the use of Laser Doppler Vibrometry (LDV) to measure out-of-plane vibrations [16,17]. This allows for the accurate evaluation of the frequency response of SAW resonators, which is essential in filter design. However, shear horizontal surface acoustic waves (SH-SAWs) [18] often exhibit no out-of-plane components within the wave aperture, and thus require unconventional detection approaches. Shear horizontal (SH) waves, such as Bleustein–Gulyaev waves (BGWs) [19,20], are attractive in filter design [21,22], since they often offer higher quality factors, as well as in other applications [3,7,23].

For SAW devices based on semi-transparent substrates, such as lithium tantalate, one approach involves passing a laser beam through the material and observing polarization changes due to shear strain [24]. However, this method does not apply to opaque substrates or those covered with opaque films.

Another approach is to place the substrate at a tilted orientation with respect to the incident laser beam [25,26]. In this case, the beam must land on the edge of a structure erected on the piezoelectric substrate. This allows the in-plane vibration to be detected through LDV. However, the tilt angle creates challenges that increase the complexity of the experimental setup. It is also not applicable for SAWs that have few or no erect structures on the substrate surface, except for the IDT. However, the IDT thickness is usually in the sub-micrometer scale [27,28], which is small in comparison with the laser beam spot size.

Another procedure involves using multiple simultaneous laser beams [29,30], which land at different angles with respect to the substrate in order to capture both in-plane and out-of-plane motions. A similar technique utilizes a single laser beam, but changes the angle of the substrate mechanically [31]. These techniques form the basis of specialized 2D and 3D vibrometry, but come at a significantly increased experimental setup complexity and cost.

More common approaches to full-field capture rely on fast cameras [32] and 3D digital image correlation (3D-DIC) [33]. The images are analyzed to detect in-plane motion, and thus, high-speed cameras are required in high-frequency applications. This usually inflates the complexity and cost of 3D-DIC and is limited in frequency by the frame rate of the camera used.

In this paper, we present a novel approach to detecting SH-SAWs using traditional 1D vibrometers that are applicable to high-frequency SH-SAW-based devices. The proposed technique is first explained, and then finite element analysis (FEA) is used for initial verification. Finally, experimental validation is presented by detecting an SH-SAW, namely a BGW.

## 2. Proposed Technique

SH waves propagate within the aperture, which is set by the dimensions of the IDTs. The aperture of the wave is confined to the area where the IDT fingers overlap. Typically, the overlap length is assumed to be large enough for the wave to be considered invariant along that direction. Beyond this aperture, the behavior is usually not of interest to researchers and engineers.

However, just outside of the wave aperture, at the fingertip, there is an area that can be of great value. SH waves act as a boundary load on this area and result in out-of-plane surface strains and deformations. Since this deformation is directly related to the SH wave’s time history, it is ripe for exploitation as a measure of the SH wave. We propose the use of LDV to measure those deformations by using an incident laser beam focused on this area.

**Figure 1:**
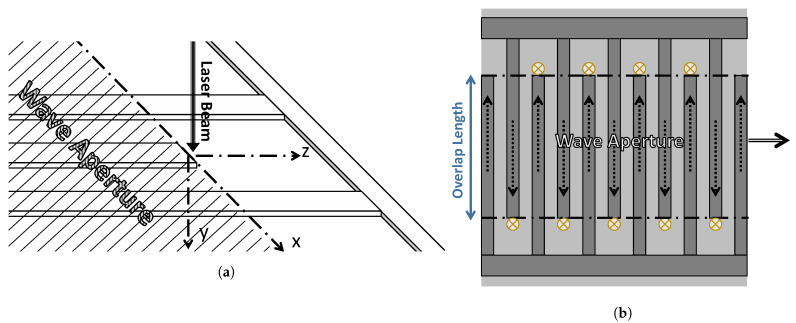
(**a**) The experimental configuration of the proposed measurement technique. (**b**) Positions of the laser spot (marked by ⊗), the displacement field u(*x*, *y*, *z*, *t*) (marked by the dashed-line arrows), and the propagation direction of the shear horizontal (SH) wave (marked by the double-line arrow).

Figure 1a shows a schematic of the proposed experimental configuration. The origin of the coordinate system is taken on the surface of the substrate directly below the intersection of the finger’s mid-line and its tip. The wave propagates along the x-axis, creating a displacement field u(x,y,z,t) along the z-axis. A laser beam that is incident along the y-axis is used to measure the out-of-plane surface deformation $w(0,0,z_{\circ },t)$ at the point on the z-axis where the deformation reaches a maximum.

Figure 1b shows the top view of the IDT, the SH wave, and the allowable locations of the laser beam spot on the substrate. Any of those positions can be used in the proposed detection scheme. To characterize the displacement field u(x,y,z,t), we need to identify a relationship between the time history of the measurement signal $w_{\circ }\left(t\right)=w\left(0,0,z_{\circ },t\right)$ and the instantaneous amplitude of the shear wave $u_{\circ }\left(t\right)$. We hypothesize the existence of a constant relationship such that $\alpha =w_{\circ }/u_{\circ }$. The FEA software COMSOL was used to test the validity of this hypothesis and to estimate α.

## 3. FEA

**Figure 2:**
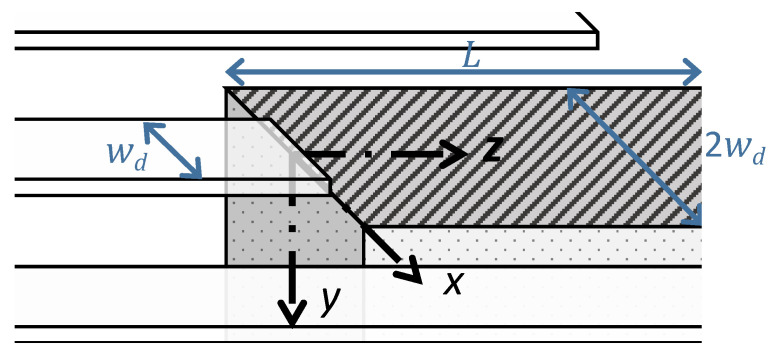
Interdigitated electrode (IDT) fingers and the piezoelectric substrate. The highlighted section represents the sector under study.

The region under study was a cuboid sector of the substrate bounded by the fingertip and the substrate edge (Figure 2). The distance between those boundaries was on the order of *L* = 2 mm. The finger width was $w_{d}$ = 10 μm and the total width of the region under study was $2w_{d}$, with a margin of $\frac{1}{2}w_{d}$ on either side of the finger. The substrate was made of Navy Type I Lead Zirconate Titanate I (PZT4) with a Young’s modulus of E=63 GPa and a Poisson ratio of ν=0.32. The thickness was set to H=3 mm to match that of the fabricated substrate. This region was meshed in the Finite Element Model (FEM) using 60,344 tetrahedral elements with 20,230 mesh vertices.

By assuming a wave frequency that is away from structural resonances of the sector under study, we could analyze its quasi-static response under the in-plane displacement:

(1) $$u\left(x,y,0\right)=u_{\circ }e^{-\frac{\pi }{\lambda r}y};\;-\frac{1}{2}w_{d}\leq x\leq \frac{1}{2}w_{d}\;\;\&\;\;0\leq y\leq H.$$

This was applied by the finger to the left face (z=0) of the sector, where $u_{\circ }=u_{\circ }\left(t\right)$ is the instantaneous value of the displacement field at the fingertip, λ is the wavelength, and *r* is the wave decay rate. To preclude rigid body motions, the bottom plane of the substrate was held fixed at u(x,H,z)=0, while the substrate faces at y=0, $x=\pm w_{d}$, and z=L were left free.

**Figure 3:**
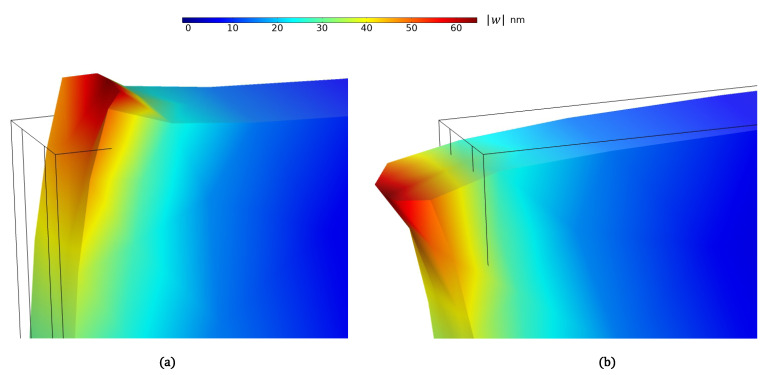
Finite element analysis (FEA) of the deformation under (**a**) positive and (**b**) negative in-plane displacement, *u*.

Figure 3 shows the resulting surface deformation under compressive (positive) and tensile (negative) prescribed in-plane displacements of $u_{\circ }=\pm 100$ nm, where the wavelength and decay rate were set to λ=40 μm and r=1. Varying the prescribed displacement $u_{\circ }$ within this range, the relationship between $u_{\circ }$ and the peak surface deformation $w_{\circ }=w\left(0,0,z_{\circ }\right)$ was found to be linear.

**Figure 4:**
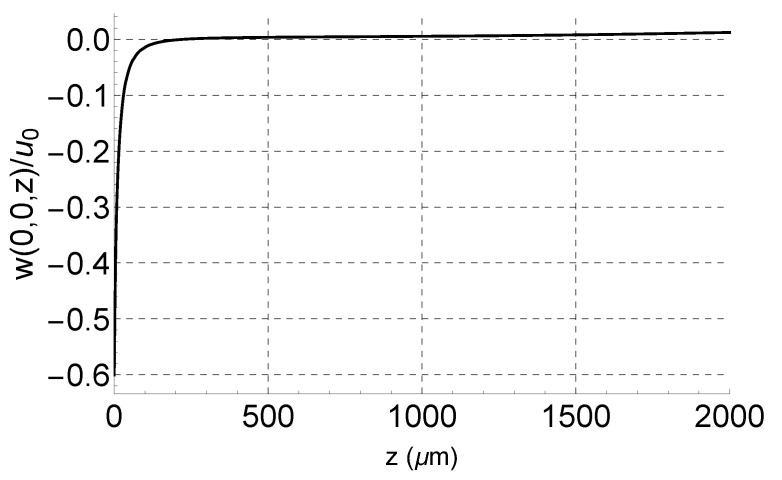
The normalized surface deformation simulated by COMSOL.

We investigated the variation in the surface deformation along the mid-line of the finger (z-axis) beyond the tip w(0,0,z) in order to determine the maximum point’s offset distance from the fingertip $z_{\circ }$ and, therefore, the optimal location of the laser spot. Figure 4 shows w(0,0,z) normalized with respect to the maximum in-plane displacement $u_{\circ }$. The out-of-plane displacement decays exponentially away from the fingertip, and therefore, the optimum position to place the laser beam is at the fingertip with $z_{\circ }=u_{\circ }$.

Most SH waves have sub-micron shear displacements, while the LDV laser spot is typically between 1 and 10μm. Therefore, the translation of the fingertip would not interrupt measurement, and the laser spot can keep the optimal point $z_{\circ }$ within focus. However, achieving optimal positioning would require an infinitesimal spot size. Since the spot size is finite and the deformation field decays exponentially, we propose the location of the spot in direct “contact” with the fingertip.

**Figure 5:**
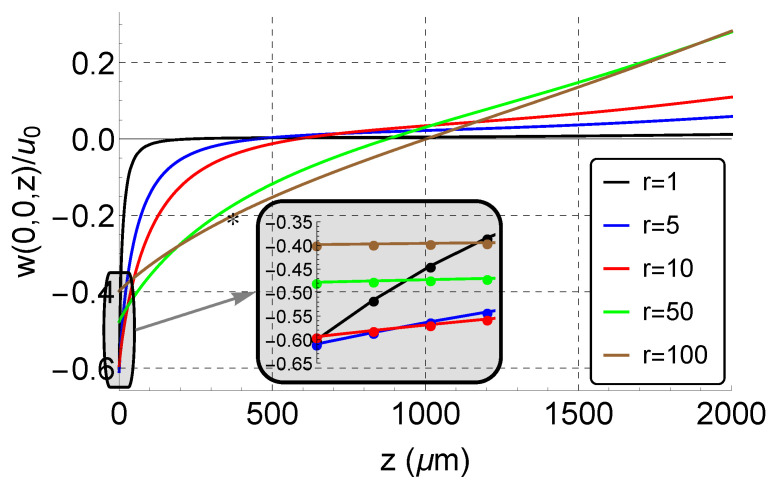
Variation in the normalized surface deformation as a function of surface wave confinement.

The decay rate of the deformation field depends on the type of SH-SAW, the substrate properties, and the boundary conditions. This rate determines the degree of the SH wave’s localization to the substrate surface. To determine whether variation in the decay rate may impose further limits on the proposed experimental technique, we compare in Figure 5 the normalized deformation along the fingertip mid-line $w\left(0,0,z\right)/u_{\circ }$ for a range (*r* = 1–100) of decay rates. It was found that the surface deformation resulting from less-confined shear waves decays at a slower rate. For r=100, the surface deformation drops to half value at z=370 μm, which is marked with an asterisk in Figure 5. The linear relationship between the peak in-plane displacement $u_{\circ }$ and the peak surface deformation $w_{\circ }$ also changes from $w_{\circ }=-0.6u_{\circ }$ at r=1 to $w_{\circ }=-0.4u_{\circ }$ at r=100. Highly confined surface waves exhibit a higher deformation ratio α, but require a smaller laser spot.

As expected, the relationship between in-plane displacement and out-of-plane deformation was found to be independent of Young’s modulus and linearly dependent on Poisson’s ratio. Specifically and for a decay rate of r=1, the deformation ratio varied from α=−0.45 for ν=0.1 to α=−0.63 for ν=0.5.

## 4. Experimental Validation

The proposed technique was deployed to characterize BGWs. Such waves [19,20] propagate exclusively on the surface of shear-poled piezoelectric materials. They are composed of a shear displacement field u(x,y,z) along the *z*-direction (Figure 1a) coupled to an electromagnetic field propagating in the *x*-direction. BGWs have been used in telecommunication filters [7] and viscosity sensors [34,35,36,37].

**Figure 6:**
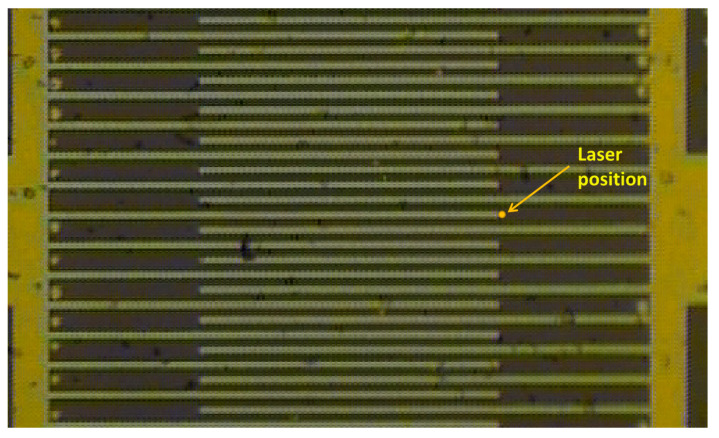
A microscopic image of the fabricated Bleustein–Gulyaev wave (BGW) IDT showing the location of the Laser Doppler Vibrometry (LDV) laser spot.

A 64 MHz BGW resonator was designed and fabricated by patterning an IDT on the surface of a shear-poled PZT4 substrate. The width of the fingers was set to 10 μm, corresponding to a wavelength of λ=40 μm. The IDTs were fabricated on a polished and shear-poled PZT4 substrate. Using physical vapor deposition, a 400 nm thick aluminum layer was sputtered onto the substrate. A positive photoresist (Shipley 1811) was spun onto the substrate, baked, patterned using lithography, and developed using MF-319. Finally, an aluminum etchant was used to etch the aluminum layer into the desired electrode pattern, and the photoresist was stripped. Figure 6 shows the fabricated resonator under the microscope.

The behavior of BGWs in PZT4 is well documented [7,38]. The response to a potential field imposed by applying voltage waveform V(t) to the substrate surface via an IDT is the displacement field [39]:

(2) $$u_{\circ }\left(t\right)=d_{15}V\left(t\right),$$

where $d_{15}=0.496$ nm/V [38] is the shear stress piezoelectric constant. This relationship can be expressed in terms of the measured out-of-plane deformation as:

(3) $$w_{\circ }\left(t\right)=0.496\alpha V\left(t\right)\left(\mathrm{nm}\right).$$

The resonator was placed under the microscope-based Polytech MSA-600 Laser Doppler Vibrometer. Direct probing of the IDT under the microscope was employed to supply the drive voltage V(t). BGWs were excited quasi-statically via a ramp waveform with a frequency of 30 kHz—far away from resonance—using a B&K Precision-4054 function generator.

## 5. Results and Discussion

**Figure 7:**
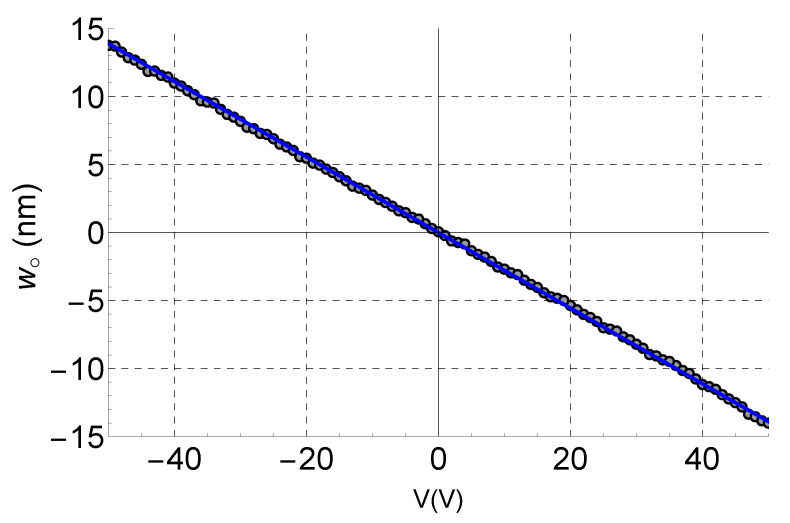
Experimental validation for the proposed technique. Experimental measurements are shown as black circles, and the model prediction line is shown as a solid blue line.

The LDV laser beam spot was located directly in front of the fingertip (Figure 6). The measured response to the rising side of the ramp was averaged 30 times. Figure 7 compares the experimental results (black circles) to the values predicted by Equation (3) (blue line), where the displacement ratio was set to α=−0.56.

Assuming that the vibrometer-measured displacement represents the average surface deformation within the field illuminated by the spot, Figure 5 can be used to estimate the decay rate *r* given the spot size. In our case, the laser beam spot size was measured as 9 μm. An FEM simulation was then employed to identify the decay rate *r*, where the average of the deformation ratio α over the initial 9 μm along the z-axis was the same as that evaluated experimentally. It was found to be r=10. This is reasonable considering that the metallization of the IDT enhances the confinement of the wave to the substrate surface [40].

The relationship between displacement and applied voltage was found to be linear for PZT4, as shown in Figure 7. This is in agreement with the literature on the small-displacement response of piezoelectric materials [41], thereby further validating the proposed technique. It is also in agreement with the linear relationship (Equation (3)) predicted through FEA.

The proposed technique can, therefore, be used to characterize the instantaneous amplitude of the shear displacement $u_{\circ }\left(t\right)$ of SH-SAWs. It can also be used to estimate wave confinement *r* to the surface. As a result, it can be used to characterize the time history and frequency response of SH-wave-based sensors and actuators. However, the excitation frequency must be restricted to a frequency range away from the resonances of the substrate sector in front of the fingertip. This restriction does not represent an onerous limitation, since those resonances occur in a much higher frequency range than resonances of the IDT due to the respective sizes of the IDT and the sector.

Moreover, once the displacement ratio α has been determined under forced excitation conditions, away from the IDT resonances, the IDT can be excited at resonance in order to determine its frequency response and identify its quality factor. The quality factor can be calculated as the ratio of the response at resonance to the static response or using the half-power bandwidth method.

## 6. Conclusions

We developed a novel technique that uses a 1D Laser Doppler Vibrometer to detect and characterize shear horizontal surface acoustic waves (SH-SAWs). The technique exploits the out-of-plane deformation appearing on the boundaries of the wave aperture as it propagates within. The proposed method was analyzed using a finite element model and validated experimentally using a Bleustein–Gulyaev resonator. It was successful in estimating the in-plane displacement field and the wave decay rate. This technique, therefore, provides researchers with a quick and effective method for characterizing SH-SAWs.

## Data Availability

The data that support the findings of this study are available from the corresponding author upon reasonable request.
